# Supplementary material for: Design, optimization and validation of genes commonly used in expression studies on DMH/AOM rat colon carcinogenesis model
Source: PeerJ. 2019 Jan 29;7:e6372. doi: 10.7717/peerj.6372 (PMC6357868; doi:10.7717/peerj.6372)
Supplement: Table S1 [file peerj-07-6372-s001.docx]

| **Gene (Accession No.)** | **Primer sequences (5’-3’)** | **Reasons for discard** |
| --- | --- | --- |
| **Bcl2**  (NM_016993.1) | F:GATTGTGGCCTTCTTTGAG | PMID16085054. Specificity not demonstrated by any of gradient annealing temperatures tested by PCR. Some changes has been done in reverse primer in order to become *in silico* specific. |
|  | R: CAAACTGAGCAGAGTCTTC |  |
| **Dkk1**  (NM_001106350.1) | F:GGGAATTACTGCAAAAACGGAATA | Based on PMID[27216891](https://www.ncbi.nlm.nih.gov/pubmed/27216891). Only unspecific bands detected within annealing temperature range of 51-54.8C. |
|  | R: ATGATCGGAGGCAGACAGAG |  |
|  | F: ATGCCCTCTGACCACAGCCATT | PMID22133691. Only unspecific bands detected within annealing temperature range of 51-54.8C. |
|  | R: CACCGTGGTCATTGCCAAGGT |  |
|  | F: ATTCCAGCGCTGTTACTGTG | PMID27468227. Specificity not demonstrated by any of gradient annealing temperatures tested by PCR. |
|  | R: GAATTGCTGGTTTGATGGTG |  |
| **iNOs**  (NM_012611.3) | F: GACCAGAAACTGTCTCACCTG | PMID22260992. Primers not specific. Other bands detected in agarose gel |
|  | R: CGAACATCGAACGTCTCACA |  |
| **p53**  (NM_030989.3) | F: ATGGAGGATTCACAGTCGGATA | Based on PMID10889512. Primers not specific. Other bands detected in agarose gel |
|  | R: GACTTCTTGTAGATGGCCATGG |  |
| **Vegfa**  (ENSRNOG00000019598) | F: GTCCTGTGTGCCCCTAATGC | PMID15829992. Specificity not demonstrated by any of gradient annealing temperatures tested by PCR. |
|  | R: CGCTCTGAACAAGGCTCACAGT |  |

**S1 Table.** List of the primer pairs not validated successfully.
