# Supplementary material for: Design, optimization and validation of genes commonly used in expression studies on DMH/AOM rat colon carcinogenesis model
Source: PeerJ. 2019 Jan 29;7:e6372. doi: 10.7717/peerj.6372 (PMC6357868; doi:10.7717/peerj.6372)
Supplement: Figure S1 [file peerj-07-6372-s002.pdf]

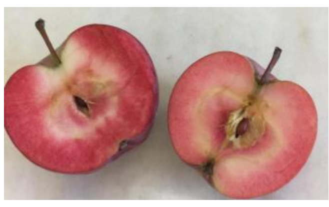

Lleida**A**pple**R**at **S**tudy (**LLARS**)

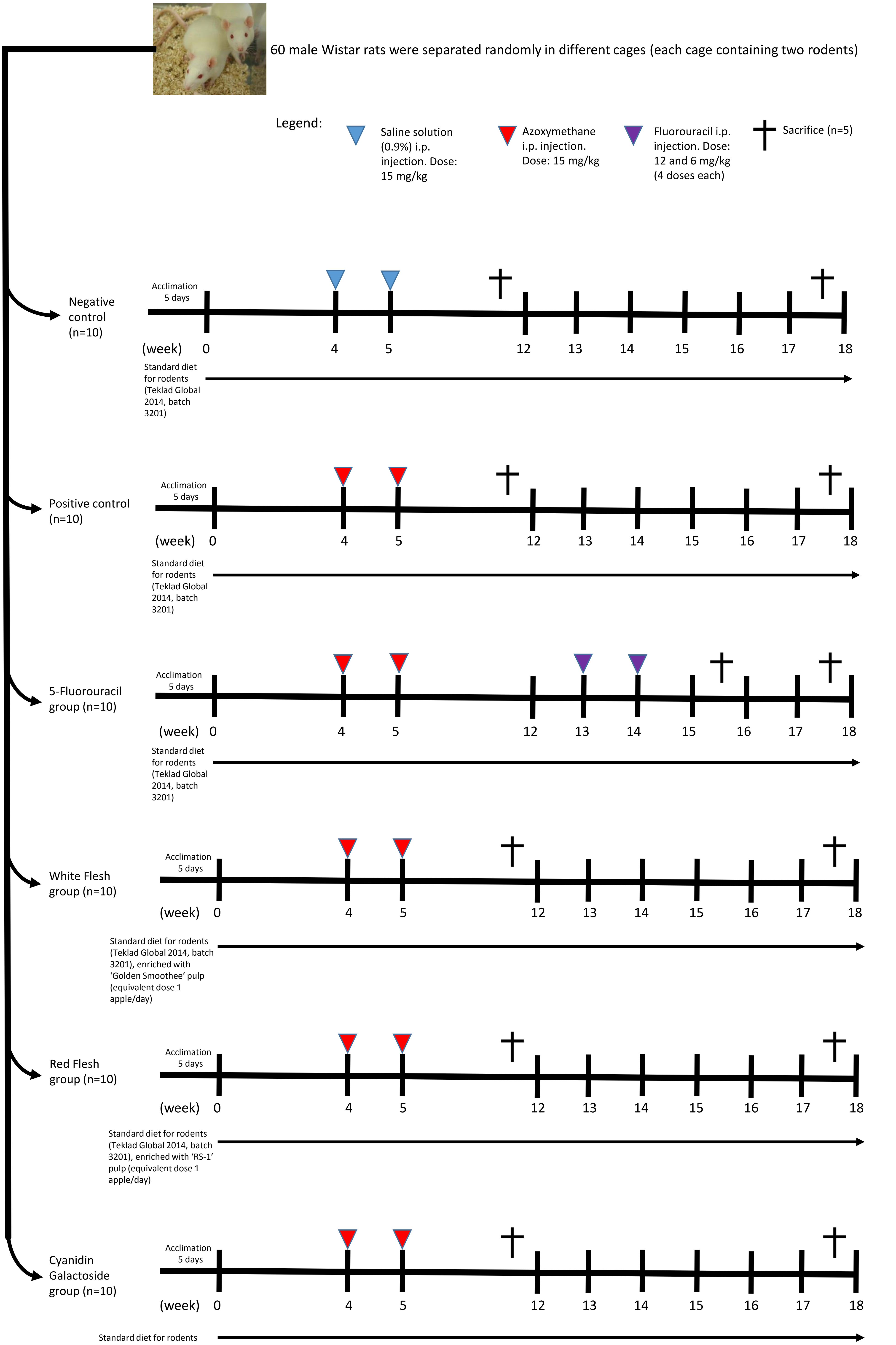

Figure S1. Long-term dietary study to evaluate the possible aptitude of anthocyanins in the early stages of colon cancer in azoxymethane (AOM) rat colon cancer model.
