# Supplementary figures and images for: Design, optimization and validation of genes commonly used in expression studies on DMH/AOM rat colon carcinogenesis model
Source: PeerJ. 2019 Jan 29;7:e6372. doi: 10.7717/peerj.6372 (PMC6357868; doi:10.7717/peerj.6372)

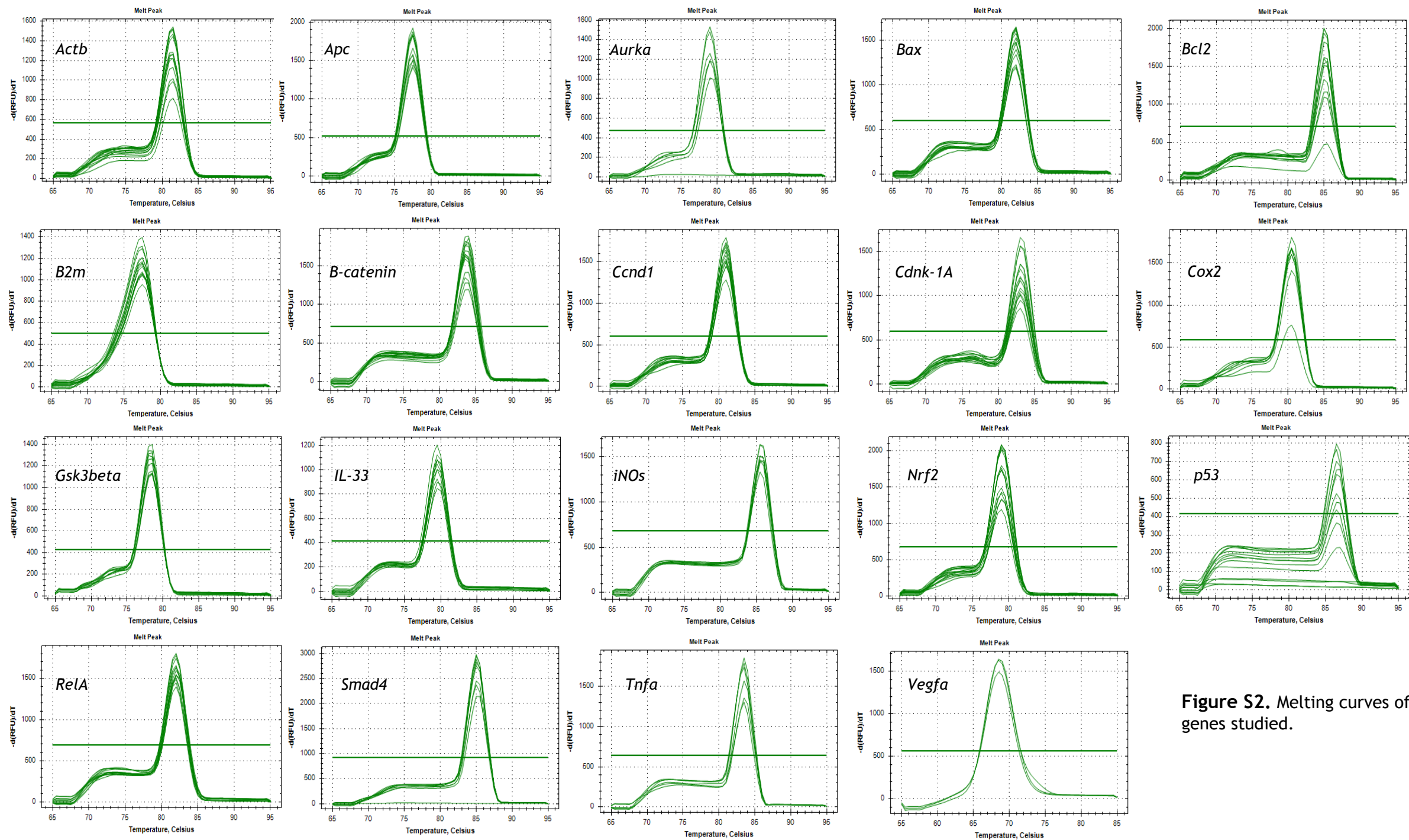

**Figure S2.** Melting curves of all genes studied.

Supplement: Figure S2 [file peerj-07-6372-s003.pdf]

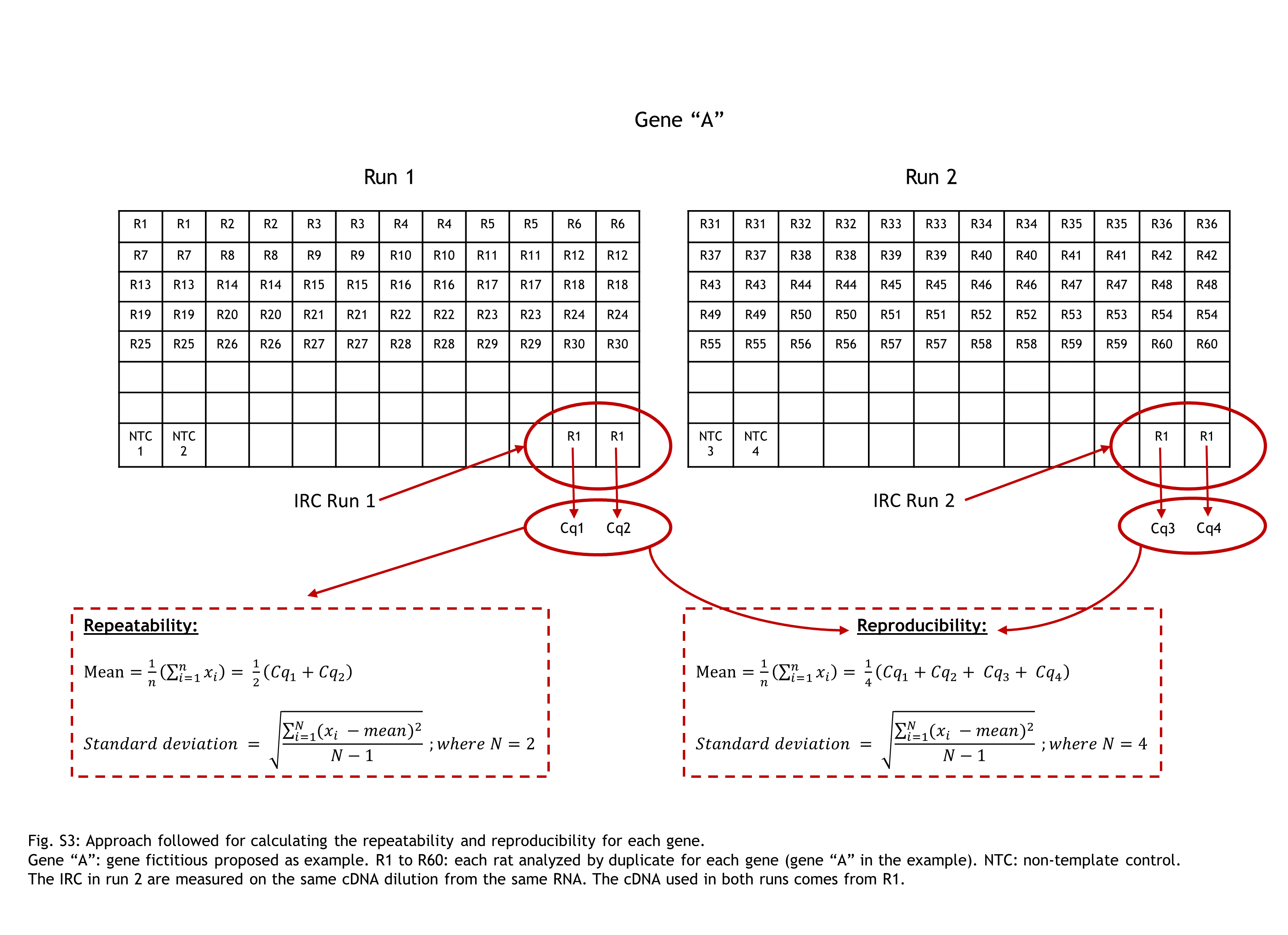

Supplement: Figure S3 — Gene “A”: gene fictitious proposed as example. R1 to R60: each rat analyzed by duplicate for each gene (gene “A” in the example). NTC: non-template control. The IRC in run 2 are measured on the same cDNA dilution from the same RNA. The cDNA used in both runs comes from R1. [file peerj-07-6372-s004.png]
