# Supplementary material for: Design, optimization and validation of genes commonly used in expression studies on DMH/AOM rat colon carcinogenesis model
Source: PeerJ. 2019 Jan 29;7:e6372. doi: 10.7717/peerj.6372 (PMC6357868; doi:10.7717/peerj.6372)
Supplement: Supplemental Information 1 [file peerj-07-6372-s005.pdf]

PDF S1

Example of agarose gel ran to check RNA integrity:

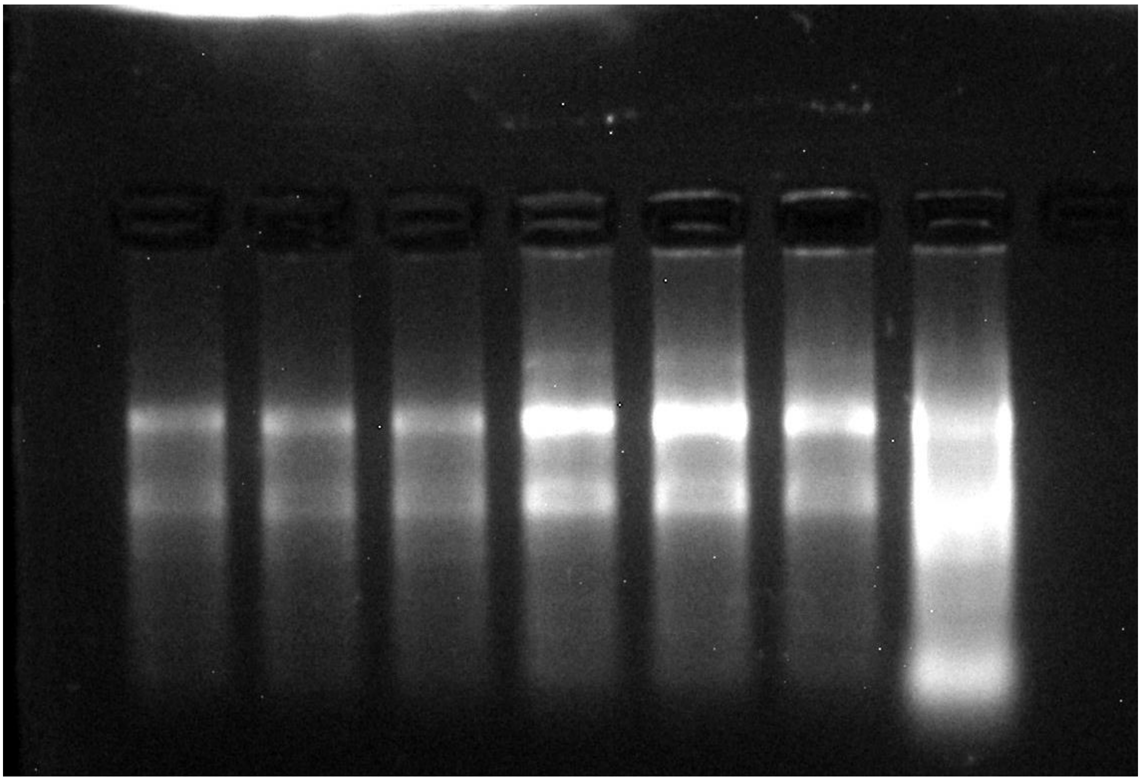

RNA electrophoresis conditions: quantity of sample/lane: 2 µl RNA + 3 µl orangine dye. TBE 0.5X. Agarose 1%. Voltage: 85 V (5V/cm). Time : 60 min. Lane 1: Distal colon, rat 2 pool; Lane 2: Distal colon, rat 2 pool; Lane 3: Distal colon, rat 2 pool; Lane 4: Ascending colon, rat 2 pool; Lane 5: Ascending colon, rat 2 pool; Lane 6: Ascending colon, rat 2 pool; Lane 7: Kidney sample "aged" (with a purpose to try to see "smear" behaviour).

RNA electrophoresis showed the two rRNA subunits depicted (18S and 28S). The upper band (corresponding to 28S) is approximately double intense than the lower band (18S). Hence, we obtained a good RNA quality, optimum for cDNA synthesis.

The lanes containing ascending colon RNA, a major intensity was obtained. This was due to that the corresponding Nanodrop values obtained: 186,6 ng/µl for distal colon and 423,6 ng/µl for ascending colon.
