# Supplementary material for: Design, optimization and validation of genes commonly used in expression studies on DMH/AOM rat colon carcinogenesis model
Source: PeerJ. 2019 Jan 29;7:e6372. doi: 10.7717/peerj.6372 (PMC6357868; doi:10.7717/peerj.6372)
Supplement: Supplemental Information 2 [file peerj-07-6372-s006.pdf]

PDF S2

Some figures showing the results of the experiments performed:

Sample of some electrophoresis performed:

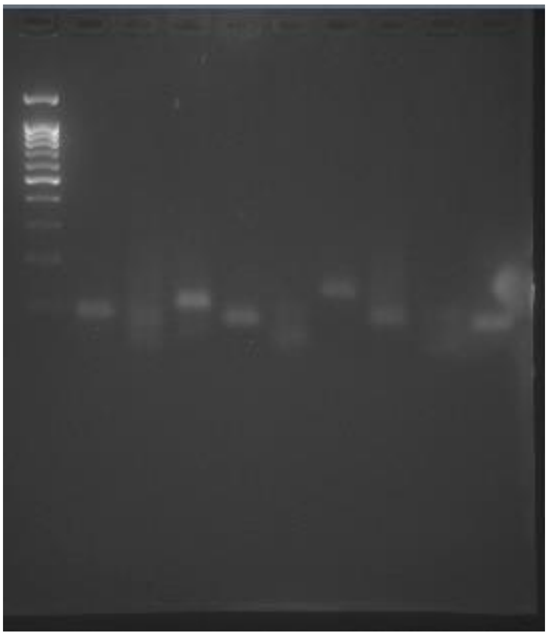

Figure 1. Electrophoresis 2% agarose, 100V, 70 min. Lane 1: DNA ladder (100, 200, 300, 400, 500, 600, 700, 800, 900, 1000, 1500 bp). Lane 2: Gsk3beta gene. Amplicon: 95 bp. Lane3: Dkk1 gene. Amplicon: 80 bp. Lane 4: APC gene. Amplicon 114 bp. Lane 5: IL-33 gene. Amplicon 84 bp. Lane 6: AURKA gene. Amplicon 98 bp. Lane 7: VEGF alpha gene. Amplicon 141 bp. Lane 8: CDNK gene. Amplicon 90 bp. Lane 9: Ccnd1 gene. Amplicon 94 bp. Lane 10: ACTB gene. Amplicon 80 bp.

The lanes highlighted with red are not specific.

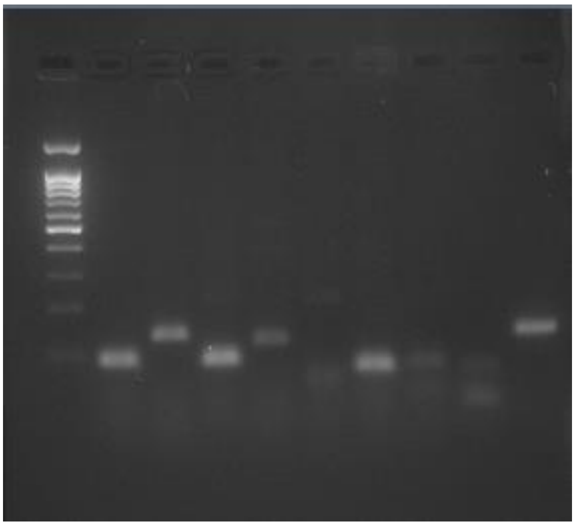

Figure 2. Electrophoresis 2% agarose, 100V, 70 min. Lane 1: DNA ladder (as previous image). Lane 2: BAX gene. Amplicon 93 bp. Lane 3: p53 gene. Amplicon 138 bp. Lane 4: Beta catenin gene. Amplicon 93 bp. Lane 5: COX2 gene. Amplicon 127 bp. Lane 6: iNOs gene. Amplicon 220 bp. Lane 7: RelA gene. Amplicon 81 bp. Lane 8: Tnf alpha gene. Amplicon 89 bp. Lane 9: Bcl2 gene. Amplicon 81 bp. Lane 10: B2M gene. Amplicon 157 bp.

The lanes highlighted with red are not specific.

For some genes an annealing gradient PCR was performed in order to choose the best Ta. An example for *β-catenin* and *Smad4* gene are showed.

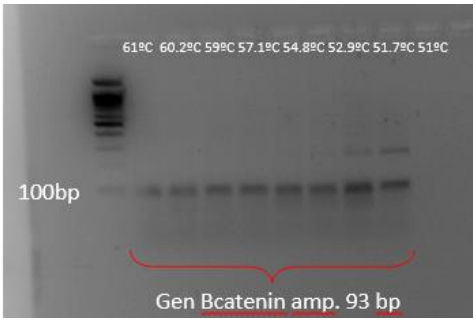

Figure 3. Electrophoresis 2% agarose, 100V, 70 min. β-catenin gene presented unspecific bands when we working with annealing temperatures of 51 – 52ºC.

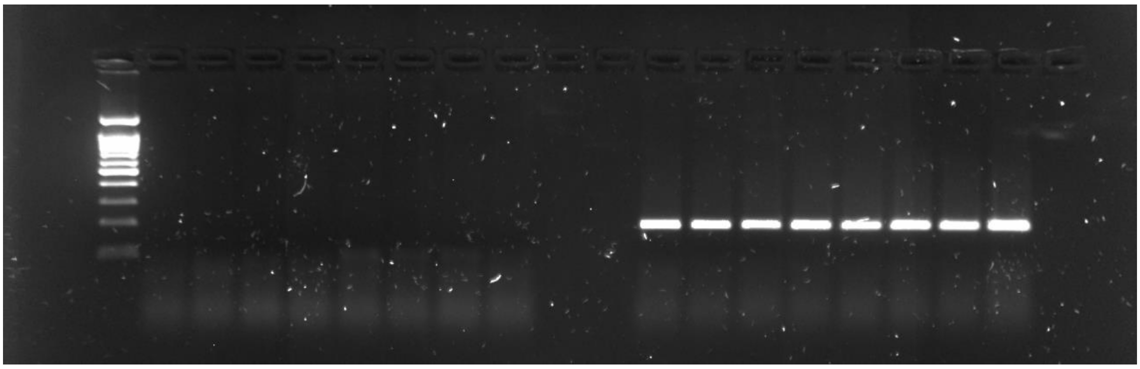

Figure 4. Electrophoresis 2% agarose, 100V, 70 min. On the left side there is an annealing temperature gradient for Dkk1 gene (from PMID27468227) where no band is detected and on the right for Smad 4 (amplicon size of 191 bp). The order of annealing temperatures in the lanes of the agarose gel are the same as **Figure 3**. In this case, Smad 4 works well in any of the annealing temperatures within the range of 51ºC to 61ºC.

Sample of some efficiency qPCR assays output:

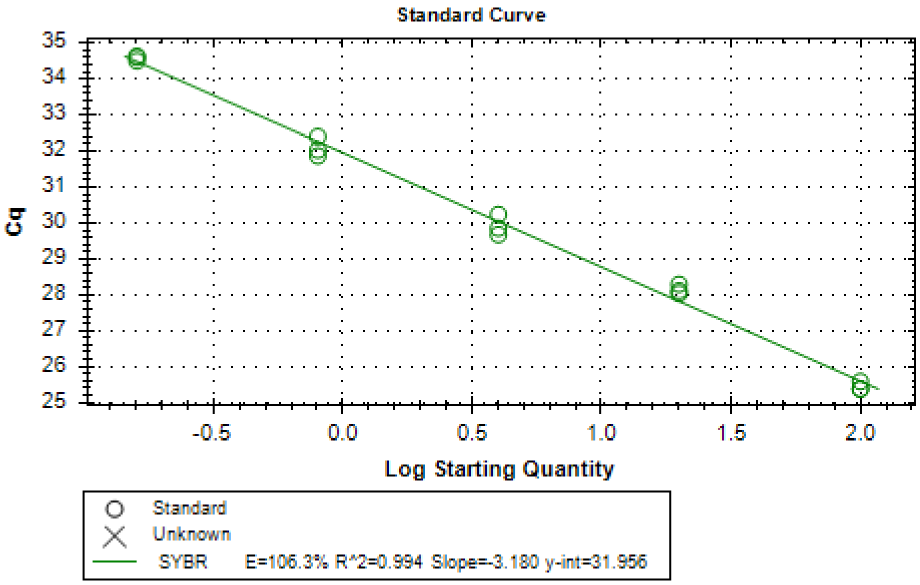

Figure 5. *BAX* gene linearity range and their % efficiency value (E).

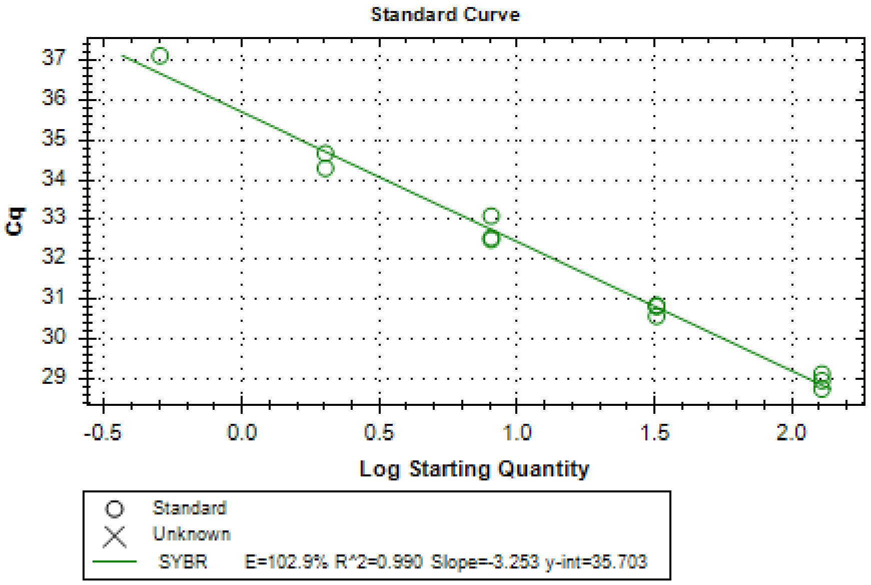

Figure 6. *Bcl2* gene linearity range and their % efficiency value (E).

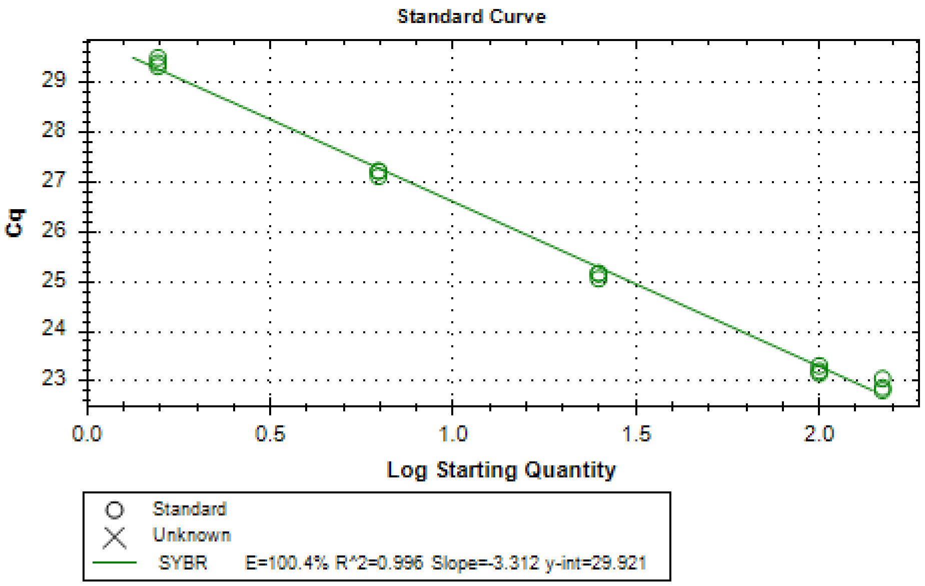

Figure 7. *B2m* gene (reference gene) linearity range and their % efficiency value (E).

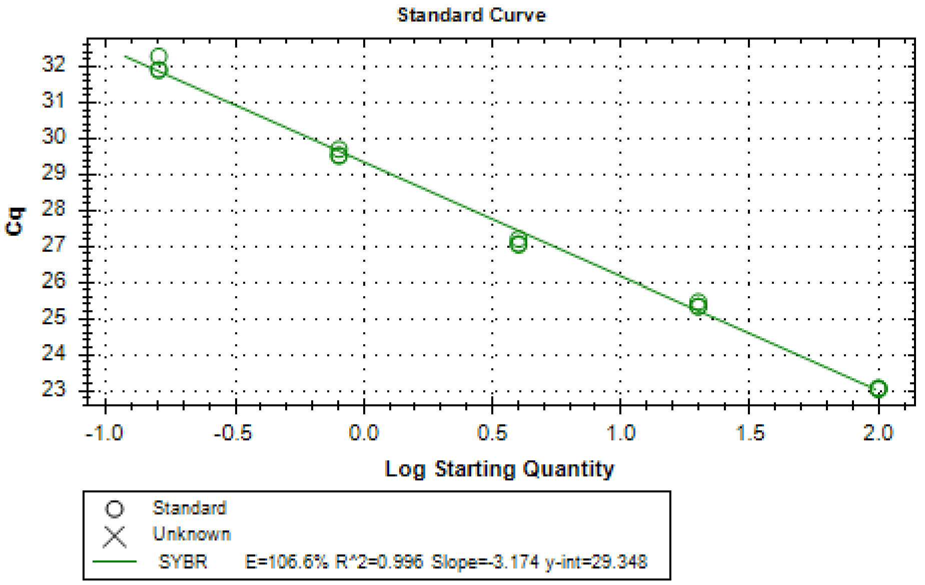

Figure 8. *Nrf2* gene linearity range and their % efficiency value (E).
