# Supplementary material for: Design, optimization and validation of genes commonly used in expression studies on DMH/AOM rat colon carcinogenesis model
Source: PeerJ. 2019 Jan 29;7:e6372. doi: 10.7717/peerj.6372 (PMC6357868; doi:10.7717/peerj.6372)
Supplement: Supplemental Information 3 [file peerj-07-6372-s007.pdf]

PDF S3

Reference gene validation (pilot scale):

	1	2	3	4	5	6	7	8	9	10	11	12	
**A**	C-1, A.1	C-1, A.2	C-1, A.3	C-2,B.1	C-2, B.2	C-2,B.3	C+1,C.1	C+1,C.2	C+1,C.3	C+2,D.1	C+2,D.2	C+2,D.3	
**B**	FU1,E.1	FU1,E.2	FU1,E.3	FU2,F.1	FU2,F.2	FU2, F.3	WF1,G.1	WF1, G.2	WF1, G.3	WF2,H.1	WF2, H.2	WF2, H.3	*Actb* gene
**C**	RF1,I.1	RF1,I.2	RF1,I.3	RF4,J.1	RF4,J.2	RF4,J.3	AE1,K.1	AE1,K.2	AE1,K.3	AE2,L.1	AE2,L.2	NTC	
**D**													
**E**													
**F**	C-1, A.1	C-1, A.2	C-1, A.3	C-2,B.1	C-2, B.2	C-2,B.3	C+1,C.1	C+1,C.2	C+1,C.3	C+2,D.1	C+2,D.2	C+2,D.3	
**G**	FU1,E.1	FU1,E.2	FU1,E.3	FU2,F.1	FU2,F.2	FU2, F.3	WF1,G.1	WF1, G.2	WF1, G.3	WF2,H.1	WF2, H.2	WF2, H.3	*B2m* gene
**H**	RF1,I.1	RF1,I.2	RF1,I.3	RF4,J.1	RF4,J.2	RF4,J.3	AE1,K.1	AE1,K.2	AE1,K.3	AE2,L.1	AE2,L.2	NTC	

Figure 1. Distal colon samples from different rats from different treatments (see Fig. S1) analysed.

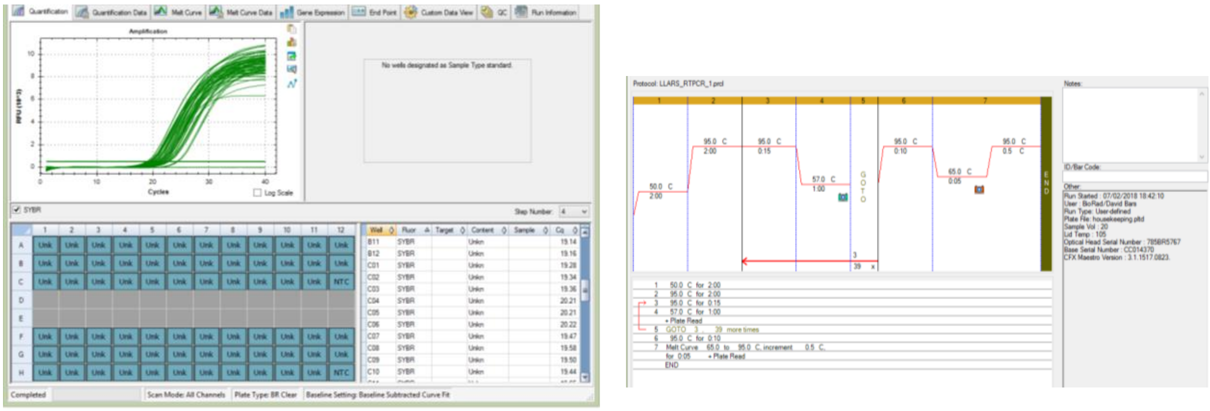

Figure 2. Output of the results obtained once analysed through Bio-Rad CFX96 real-time PCR system.

Two software resources have been used to decide if one of the two reference assayed within different experimental conditions are better or not.

Tool 1)

<u>GeNorm algorithm</u>

Bio-Rad CFX Maestro 1.1. software has a tool (based on geNorm algorithm) to check the stability (aptitude) of the candidate reference genes.

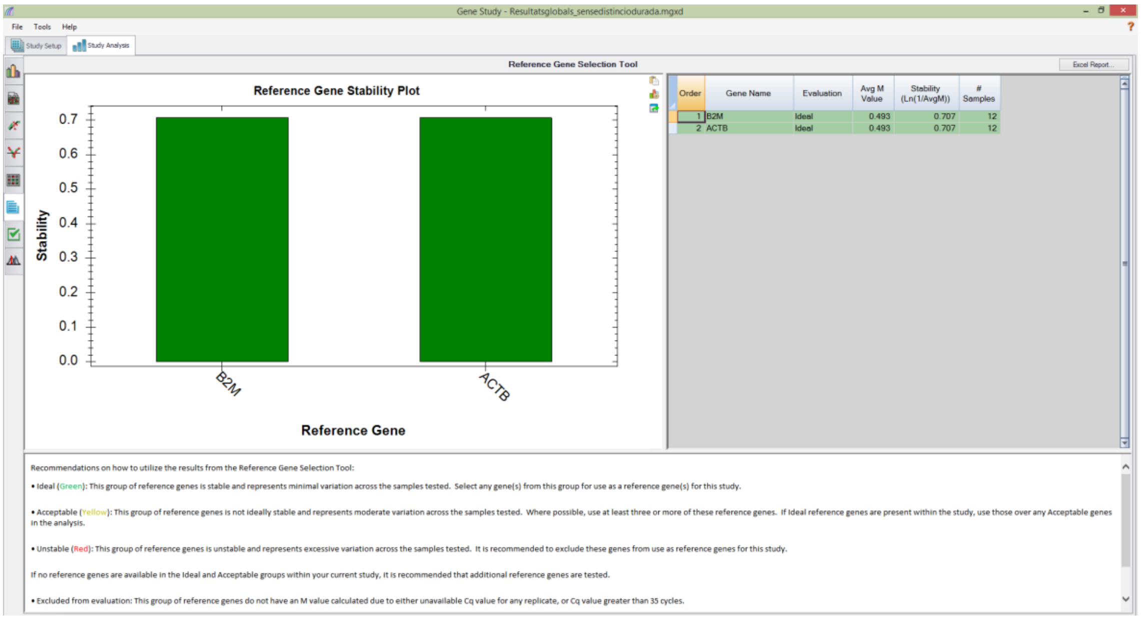

Figure 3. Output results of Bio-Rad CFX Maestro 1.1. software.

Both of them are stable along the treatments studied. Therefore, both are good candidates.

Tool 2)

Web-based RefFinder platform

http://150.216.56.64/referencegene.php?type=reference

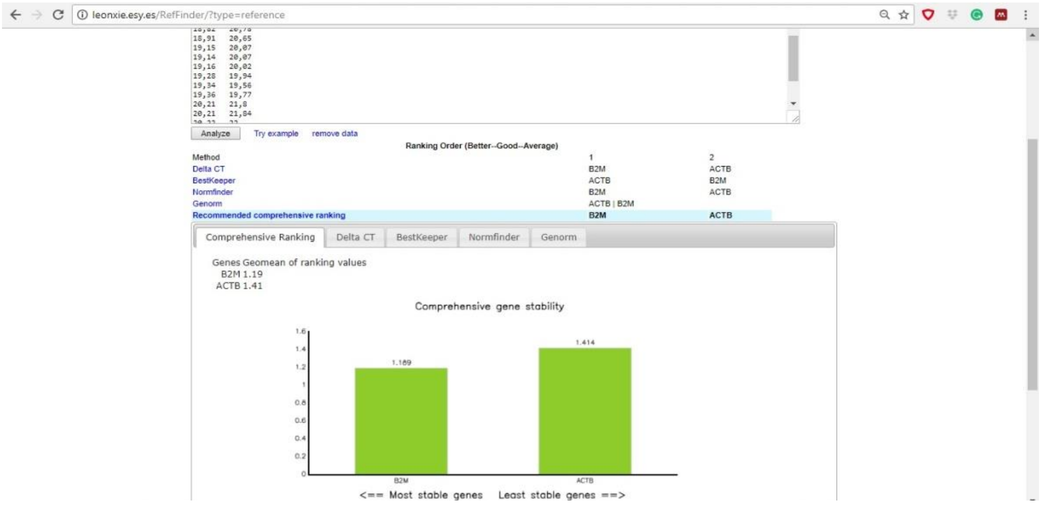

Figure 4. Output results of RefFinder platform.

RefFinder recommended us *B2M* gene instead of *Actb* gene by minor differences in geomean of ranking values, as it can be seen in the screenshot.
